# Clinically aggressive pediatric spinal ependymoma with novel *MYC* amplification demonstrates molecular and histopathologic similarity to newly described *MYCN-*amplified spinal ependymomas

**DOI:** 10.1186/s40478-021-01296-2

**Published:** 2021-12-11

**Authors:** Margaret Shatara, Kathleen M. Schieffer, Darren Klawinski, Diana L. Thomas, Christopher R. Pierson, Eric A. Sribnick, Jeremy Jones, Diana P. Rodriguez, Carol Deeg, Elizabeth Hamelberg, Stephanie LaHaye, Katherine E. Miller, James Fitch, Benjamin Kelly, Kristen Leraas, Ruthann Pfau, Peter White, Vincent Magrini, Richard K. Wilson, Elaine R. Mardis, Mohamed S. Abdelbaki, Jonathan L. Finlay, Daniel R. Boué, Catherine E. Cottrell, David R. Ghasemi, Kristian W. Pajtler, Diana S. Osorio

**Affiliations:** 1 The Division of Hematology and Oncology, St. Louis Children’s Hospital, School of Medicine in St. Louis, Washington University, St. Louis, MO USA; 2 The Steve and Cindy Rasmussen Institute for Genomic Medicine, Nationwide Children’s Hospital, 575 Children’s Crossroad, Columbus, OH 43215 USA; 3 The Division of Hematology, Oncology, Blood and Marrow Transplant, Nationwide Children’s Hospital and The Ohio State University, Columbus, OH USA; 4 Department of Pathology and Laboratory Medicine, Nationwide Children’s Hospital, Columbus, OH USA; 5 The Department of Pathology, The Ohio State University, Columbus, OH USA; 6 Division of Anatomy, Department of Biomedical Education, The Ohio State University, Columbus, OH USA; 7 The Division of Pediatric Neurosurgery, Nationwide Children’s Hospital and The Ohio State University, Columbus, OH USA; 8 The Department of Radiology, Nationwide Children’s Hospital, Columbus, OH USA; 9 The Department of Pediatrics, The Ohio State University, Columbus, OH USA; 10 Emeritus Professor of Pediatrics and Radiation Oncology, The Ohio State University, Columbus, OH USA; 11 Hopp Children’s Cancer Center Heidelberg (KiTZ), 69120 Heidelberg, Germany; 12 Division of Pediatric Neurooncology, German Cancer Research Center (DKFZ) and German Cancer Consortium (DKTK), 69120 Heidelberg, Germany; 13 Department of Pediatric Oncology, Hematology, and Immunology, University Hospital Heidelberg, Heidelberg, Germany

**Keywords:** Ependymoma, MYC, MYCN, Spinal, Amplification, FISH, DNA methylation array, Pediatric

## Abstract

Primary spinal cord tumors contribute to ≤ 10% of central nervous system tumors in individuals of pediatric or adolescent age. Among intramedullary tumors, spinal ependymomas make up ~ 30% of this rare tumor population. A twelve-year-old male presented with an intradural, extramedullary mass occupying the dorsal spinal canal from C6 through T2. Gross total resection and histopathology revealed a World Health Organization (WHO) grade 2 ependymoma. He recurred eleven months later with extension from C2 through T1-T2. Subtotal resection was achieved followed by focal proton beam irradiation and chemotherapy. Histopathology was consistent with WHO grade 3 ependymoma. Molecular profiling of the primary and recurrent tumors revealed a novel amplification of the *MYC* (8q24) gene, which was confirmed by fluorescence in situ hybridization studies. Although *MYC* amplification in spinal ependymoma is exceedingly rare, a newly described classification of spinal ependymoma harboring *MYCN* (2p24) amplification (SP-MYCN) has been defined by DNA methylation-array based profiling. These individuals typically present with a malignant progression and dismal outcomes, contrary to the universally excellent survival outcomes seen in other spinal ependymomas. DNA methylation array-based classification confidently classified this tumor as SP-MYCN ependymoma. Notably, among the cohort of 52 tumors comprising the SP-MYCN methylation class, none harbor *MYC* amplification, highlighting the rarity of this genomic amplification in spinal ependymoma. A literature review comparing our individual to reported SP-MYCN tumors (n = 26) revealed similarities in clinical, histopathologic, and molecular features. Thus, we provide evidence from a single case to support the inclusion of *MYC* amplified spinal ependymoma within the molecular subgroup of SP-MYCN.

**Supplementary Information:**

The online version contains supplementary material available at 10.1186/s40478-021-01296-2.

## Introduction

Primary spinal cord tumors are rare in children and adolescents, contributing to ≤ 10% of all pediatric central nervous system (CNS) neoplasms [52]. Intramedullary spinal ependymomas (EP) make up ~ 30% of pediatric spinal cord tumors, second to astrocytomas [52]. Histologically, spinal cord ependymomas are categorized into subependymoma (World Health Organization (WHO) grade 1), myxopapillary EP (WHO grade 2), and classic or anaplastic EP (WHO grade 2/3) [34]. Furthermore, molecular classification using DNA methylation array-based profiling distinguishes three distinct molecular subgroups of spinal EP: subependymoma (SP-SE), myxopapillary EP (SP-MP), and anaplastic EP (SP-EP) [38]. Despite these divergent molecular subgroups, spinal cord EP are typically slow growing with universally excellent overall survival rates, especially when gross total resection of the tumor can be achieved [4, 7, 33, 36, 46]. Radiation therapy is usually advocated for subtotally resected grade 2 tumors [3, 18, 32, 46] and all patients with grade 3 tumors [3, 12, 46].

Recently, a novel molecular subgroup of spinal EP with focal high-level *MYCN* (2p24) amplification was defined and found to be associated with dismal outcomes and malignant progression, despite aggressive management [20, 41, 43, 49]. This molecular pathology will be newly recognized as a distinct subgroup of spinal cord EP (SP-MYCN) in the fifth edition of the WHO Classification of Tumors of the Central Nervous System [16, 35]. Similar to SP-MP, SP-MYCN were found to develop in extramedullary spaces in sharp contrast to the intramedullary growth seen in SP-EP [20, 41, 49]. Herein, we report on an adolescent male with aggressive classic spinal EP harboring a novel focal amplification of the *MYC* oncogene located on chromosome 8q24.

## Case presentation

**Fig. 1:**
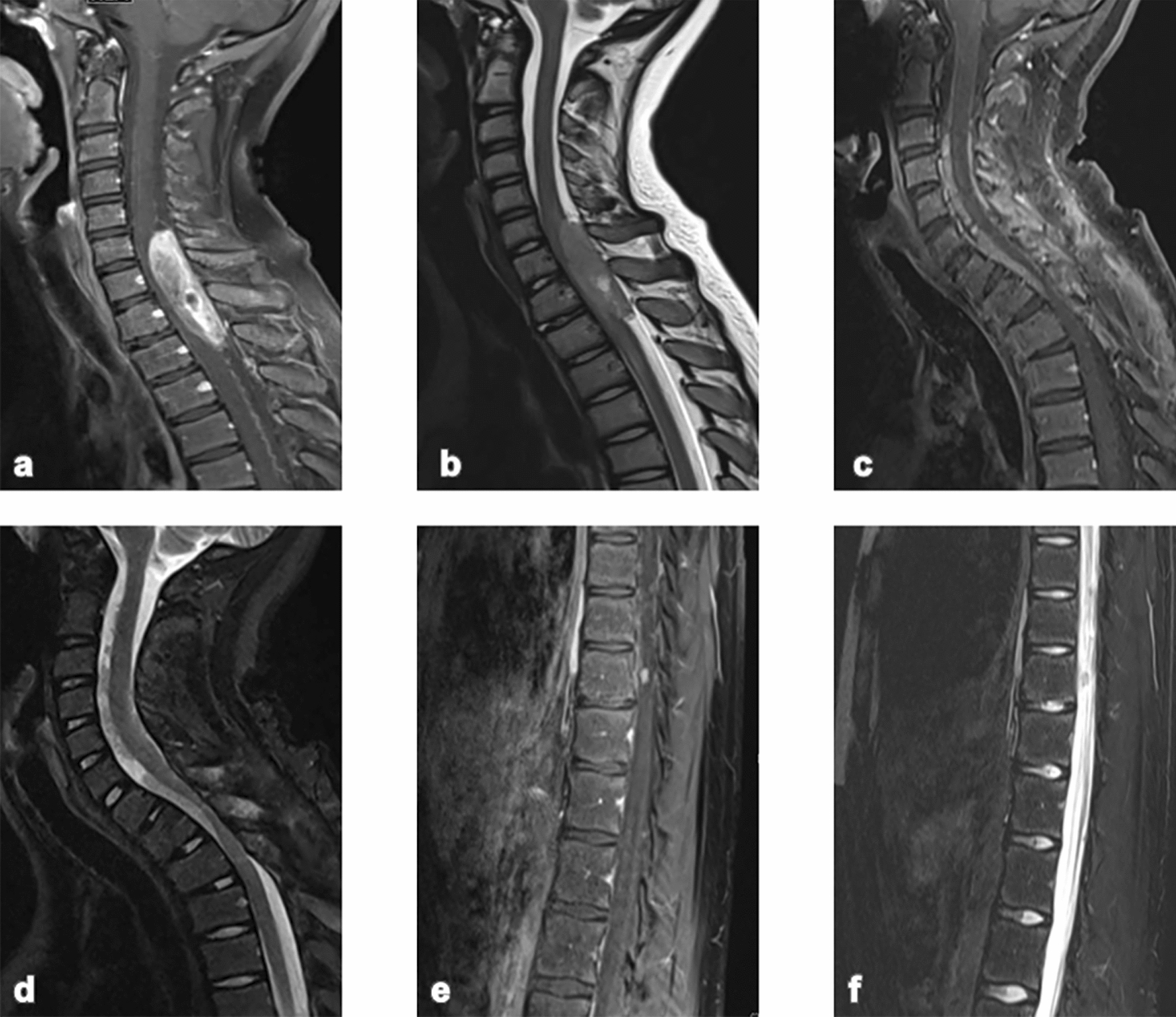
MRI images at presentation: sagittal **a** T1-weighted and **b** T2-weighted showing avidly enhancing intradural mass, occupying the dorsal spinal canal from C6 through T1-T2. MRI images at first relapse: sagittal **c** T1-weighted and **d** T2-weighted showing avidly enhancing tumor, now extending from C2 through T1-T2. MRI images at second relapse: sagittal **e** T1-weighted and **f** T2-weighted showing new noncontiguous separate nodules scattered along the surface of the cord from C7 through L1

A 12-year-old male with no significant past medical history presented to the emergency room with a three-month history of back pain and acute onset of weakness in the left lower extremity. Neurological examination was significant for left lower extremity weakness and ataxia. Magnetic resonance imaging (MRI) of the brain and spine revealed a localized avidly enhancing intradural, extramedullary mass occupying the dorsal spinal canal from C6 through T2. The tumor resulted in severe cord compression and mild edema (Fig. 1a, b). He underwent a gross total resection of the tumor followed by observation with serial imaging. Eleven months later, he re-presented with acute onset of lower extremity paresthesia and left-handed weakness. Spine MRI revealed tumor recurrence with further extension anteriorly and posterior to the cord from C2 through T1-T2 with resultant severe cord compression, again demonstrating avid enhancement (Fig. 1c, d). Management included subtotal resection of the recurrent mass, focal proton beam irradiation (50.4 Gy), followed by four cycles of chemotherapy with vincristine, etoposide, cyclophosphamide, and cisplatin, as per ACNS0831 [45], then oral vorinostat for seven months. The patient experienced further progression with distal metastases in the thoraco-lumbar region distal to his primary tumor, 16 months after first relapse (Fig. 1e, f), and completed intensity-modulated radiation therapy (IMRT) of 36 Gy to the entire thecal sac. On his post-radiation MRI, there was evidence of progression of the cervical region tumor and the patient is therefore now undergoing focal re-irradiation of the cervical region tumor. A clinical summary is presented in Table 1.

**Table 1:** Review of *MYC* and *MYCN-*amplified spinal ependymoma clinical features

Reference	Case no	Sex	Age (yrs)	Primary tumor location	*MYC* vs. *MYCN* amplification	Resection	Relapse/ Progression	Chemotherapy	Radiation therapy	Disease history
This study	1	M	12	Cervical/Thoracic	*MYC* (8q24)	GTR	Yes	Yes	Yes	Multiple recurrences, GTR of first resection at C6-T2; first recurrence at C2-T1/T2 11 months post-resection treated with STR, chemotherapy and proton therapy; progression and metastasis to thoraco-lumbar region 16 months after first relapse treated with IMRT to the entire thecal sac and focal re-irradiation to the cervical region tumor
[49]	1	F	22	Cervical	*MYCN* (2p24)	STR	No	No	Unknown	Died, cause uncertain
[49]	2	F	31	Lumbar	*MYCN* (2p24)	GTR	Yes	NA	Yes	Alive at 15- month follow-up
[49]	3	F	13	Thoracic	*MYCN* (2p24)	STR	Yes	Yes	Yes	Died of disease
[49]	4	F	15	Thoracic	*MYCN* (2p24)	NA	NA	NA	NA	NA
[20]	1	F	14	Cervical/Thoracic	*MYCN* (2p24)	STR	Yes	Yes	Yes	Dead
[20]	2	M	18	Thoracic	*MYCN* (2p24)	STR	Yes	Yes	Yes	Alive at 111-month follow-up
[20]	3	M	12	Cervical/Thoracic	*MYCN* (2p24)	STR	Yes	Yes	Yes	Dead
[20]	4	F	35	Cervical/Thoracic	*MYCN* (2p24)	STR	Yes	Yes	Yes	Alive with palliative care (29-month follow-up)
[20]	5	M	34	Lumbar	*MYCN* (2p24)	STR	Yes	Yes	Yes	Dead
[20]	6	F	26	Thoracic	*MYCN* (2p24)	STR	Yes	Yes	Yes	Alive at 31-month follow-up
[20]	7	M	23	Thoracic	*MYCN* (2p24)	STR	Yes	Yes	Yes	NA
[20]	8	F	32	Thoracic	*MYCN* (2p24)	STR	Yes	Yes	Yes	Dead
[20]	9	F	56	NA	*MYCN* (2p24)	GTR	NA	No	Yes	Dead
[20]	10	M	35	Cervical/Thoracic	*MYCN* (2p24)	STR	No	Yes	Yes	Alive at 4-month follow-up
[20]	11	M	37	Cervical/Thoracic	*MYCN* (2p24)	STR	No	No	Yes	Alive at 11-month follow-up
[20]	12	F	46	Cervical	*MYCN* (2p24)	No	Yes	Yes	No	Alive at 2-month follow-up
[20]	13	F	16	NA	*MYCN* (2p24)	NA	NA	NA	NA	Dead
[41]	1	M	52	Thoracic/Brain	*MYCN* (2p24)	NA	NA	NA	NA	Alive at 2-month follow-up
[41]	2	F	24	Cervical/Thoracic/Lumbar	*MYCN* (2p24)	NA	NA	NA	NA	Alive at 14-month follow-up
[41]	3	F	30	Thoracic	*MYCN* (2p24)	GTR	Yes	Yes	No	Alive at 17-month follow-up
[41]	4	M	36	Thoracic	*MYCN* (2p24)	STR	Yes	Yes	Yes	Alive at 55-month follow-up
[41]	5	F	37	Thoracic	*MYCN* (2p24)	Resection	Yes	No	Yes	Alive at 62-month follow-up
[41]	6	F	35	Cervical/Thoracic	*MYCN* (2p24)	STR	Yes	Yes	No	Alive at 63-month follow-up
[41]	7	M	52	Cervical/Thoracic/Lumbar	*MYCN* (2p24)	Resection	Yes	Yes	No	Dead
[41]	8	M	29	Cervical	*MYCN* (2p24)	STR	Yes	Yes	Yes	Dead
[43]	13	F	40	Spinal	*MYCN* (2p24)	NA	Yes	NA	NA	NA

*Yrs* years, *GTR* gross total resection, *STR* subtotal resection, *IMRT* intensity-modulated radiation therapy, *M* male, *F* female, *NA* not available

**Fig. 2:**
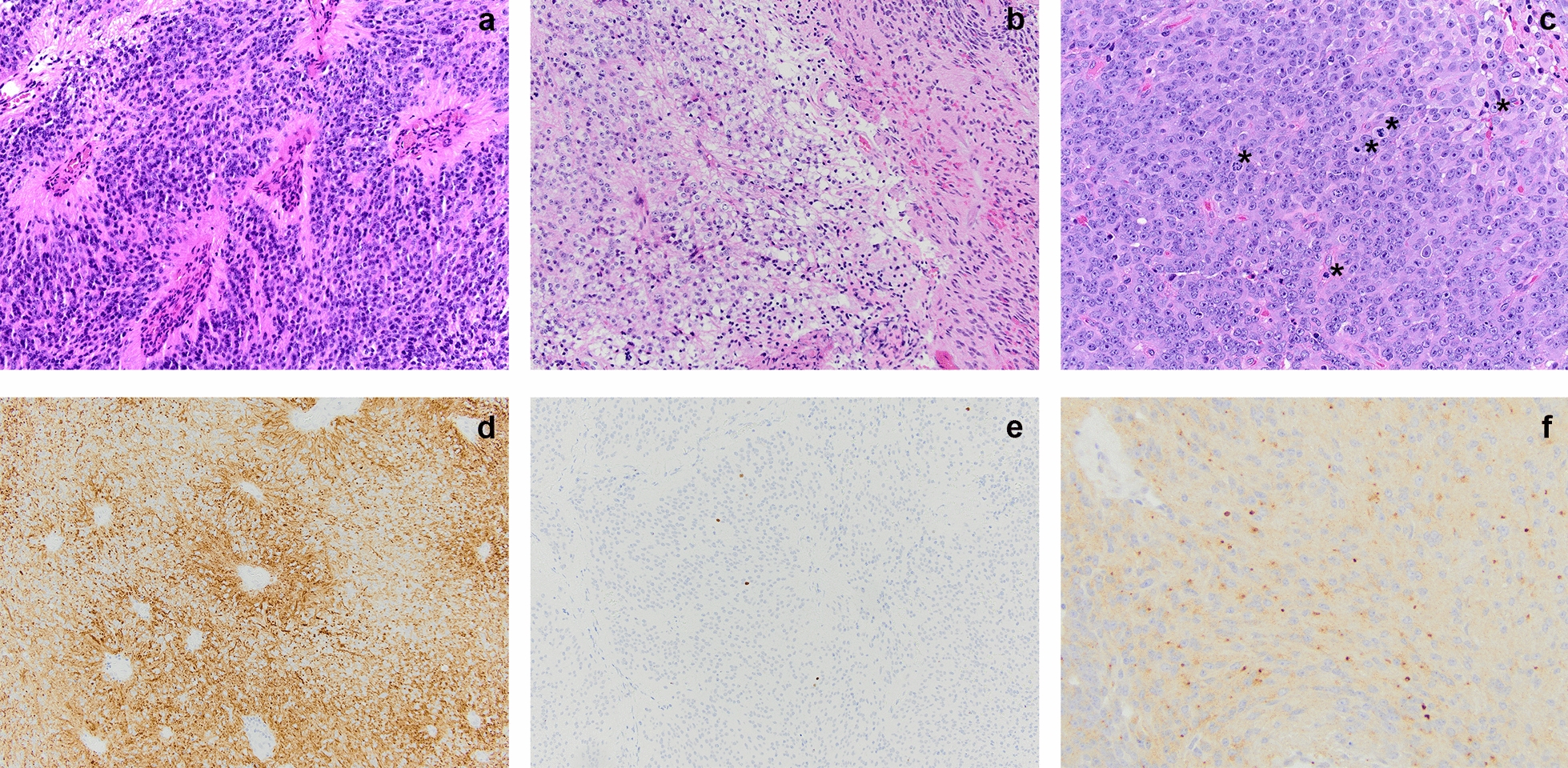
Histologic features of the primary tumor assessed by routine Hematoxylin and Eosin stain demonstrating perivascular pseudo-rosettes surrounding capillaries showing **a** microvascular proliferation (20× magnification), with **b** focal clear cell ependymoma (20× magnification). **c** Mitotically active cells are denoted by the asterisk (*) (40× magnification). The tumor cells demonstrated **d** strong perivascular GFAP (20× magnification), **e** sparse Olig-2 nuclear staining (20× magnification), and **f** dot-like EMA reactivity (40× magnification). The histopathologic findings were similar in the primary and recurrent tumors

Histopathologic examination of the initial resection specimen revealed a compact lobulated glial neoplasm with variable morphology. The majority of the tumor showed classic ependymoma features, including mildly pleomorphic tumor cells with round to oval nuclei arranged in perivascular pseudo-rosette (nuclear-free zone) formations, often surrounding capillaries showing microvascular proliferation (Fig. 2a). Other tumor areas showed clear cell change (Fig. 2b) or tanycytic morphology with elongate spindle cells. Densely cellular nests of mitotically active cells displaying conspicuous nucleoli were also present (Fig. 2c). Mitoses were counted at 3–4 per 10 high power fields (HPF). Squash preparations showed a branching angiocentric pattern of tumor cells (Additional File 1: Fig. S1a). Immunohistochemical stains demonstrated prominent perivascular glial fibrillary acidic protein (GFAP) staining (Fig. 2d) while Olig-2 nuclear staining was rare (Fig. 2e). Epithelial membrane antigen (EMA) showed perinuclear dot-like immunoreactivity (Fig. 2f), while ring-like staining was much less frequent. The Ki-67 proliferation index was focally up to 20% (Additional File 1: Fig. S1b). A diagnosis of ependymoma, WHO grade 2, with histopathologic features bordering between WHO grade 2 and WHO grade 3. The subsequent recurrent/residual tumor specimen showed similar histologic features, with even more extensive regions of hypercellular tumor and higher mitotic index (up to 8 per 10/HPF) diagnostic of anaplastic ependymoma, WHO grade 3 based on the 2016 WHO Classification.

**Fig. 3:**
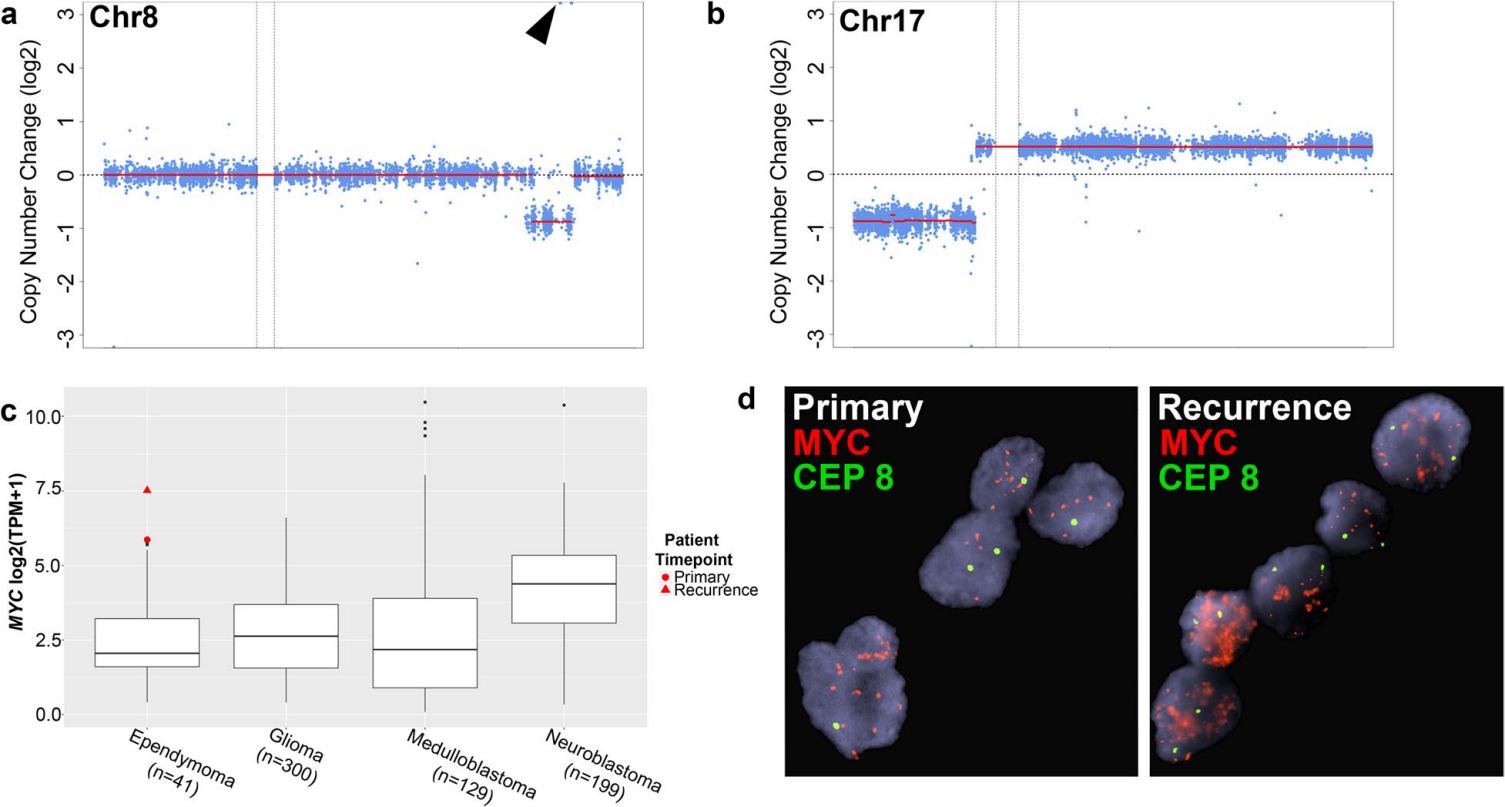
Somatic copy number alterations (CNA) on chromosome 8 (**a**) and chromosome 17 (**b**) are shown as derived from enhanced exome sequencing data. The blue points represent log2 values based on sequence depth in 100 bp windows. The red line indicates segmented CNA calls. The *MYC* (8q24) amplification is highlighted by the arrow. **c** Distribution of *MYC* gene expression in ependymomas (n = 41), gliomas (n = 300), medulloblastomas (n = 129), and neuroblastoma (n = 199) amid the UCSC Treehouse cohort and patients enrolled on our translational cancer protocol with the red points indicating our described patient case. The shape indicates timepoint (circle = primary tumor, triangle = recurrent tumor). **d** Fluorescence in situ hybridization (FISH) of the *MYC* locus (red) demonstrates gene amplification (> 20 signals compared to chromosome 8 centromere in green) with signal pattern most consistent with double minute formation

To further characterize the tumor, the patient was consented on an Institutional Review Board-approved translational research protocol and underwent comprehensive molecular profiling, including paired tumor/normal enhanced exome sequencing (eES) and RNA-sequencing (Additional File 1: Materials and Methods). This analysis included an evaluation of small single nucleotide variants (SNV), small insertion-deletions, copy number alterations (CNA), gene fusions, and aberrant gene expression. We sequenced disease-involved tissue from both the primary spinal cord tumor and localized tumor recurrence occurring 11 months after the primary tumor. We did not identify any cancer- or disease-associated SNVs or CNAs from the germline comparator peripheral blood. In addition, we did not identify any clearly medically meaningful somatic SNVs, small indels, or gene fusions. In both analyzed timepoints, the CNA profile was notable for a focal amplification of the *MYC* gene on 8q24 (Fig. 3a) and biallelic loss of 17p, including *TP53* and likely consistent with an isochromosome 17q (Fig. 3b, Additional File 1: Table S1). Other CNA described from eES included segmental biallelic losses of 8q, 10q (including *PTEN*), and 19q (Additional File 1: Fig. S2, Additional File 1: Table S1). The University of California Santa Cruz (UCSC) Treehouse Initiative (https://treehousegenomics.ucsc.edu/explore-our-data/) is a collaborative data sharing initiative whereby RNA-sequencing data from a breadth of tumor types are publicly available. We utilized a cohort of pediatric and adolescent/young adult central and peripheral nervous system tumors (n = 563) from the UCSC Treehouse Initiative to compare *MYC* gene expression. Consistent with the identified gene amplification, *MYC* was found to be overexpressed in both primary (log2 fold change: 3.95, *P* = 0.0007) and recurrent (log2 fold change: 4.57, *P* = 9.57 × 10^–5^) tumors. Visualization of *MYC* expression for our described patient case relative to ependymoma (n = 41), glioma (n = 300), medulloblastoma (n = 129), and neuroblastoma (n = 199) patients from the UCSC Treehouse Initiative and our internal cohort confirmed this overexpression (Fig. 3c).

Fluorescence in situ hybridization (FISH) of the *MYC* (8q24) and *MYCN* (2p24) loci was performed from formalin-fixed paraffin-embedded tissue to confirm the presence of *MYC* amplification. Greater than 20 copies of *MYC* were detected relative to a 2-copy state for the centromere of chromosome 8 with the amplification signal pattern most consistent with double minute formation (primary tumor: nuc ish(MYC amp)[74/100]; recurrent tumor: nuc ish(MYC amp)[92/100]) (Fig. 3d). In comparison, the *MYCN* locus was present at a 2-copy state with two signals detected for both *MYCN* and the centromere of chromosome 2 (primary tumor: nuc ish(MYCNx2)[89/100]), recurrent tumor: nuc ish(MYC amp)[87/100]) (Additional File 1: Fig. S3). These results provide orthogonal confirmation of next generation sequencing data supporting the identification of a spinal ependymoma harboring a novel *MYC* amplification.

**Fig. 4:**
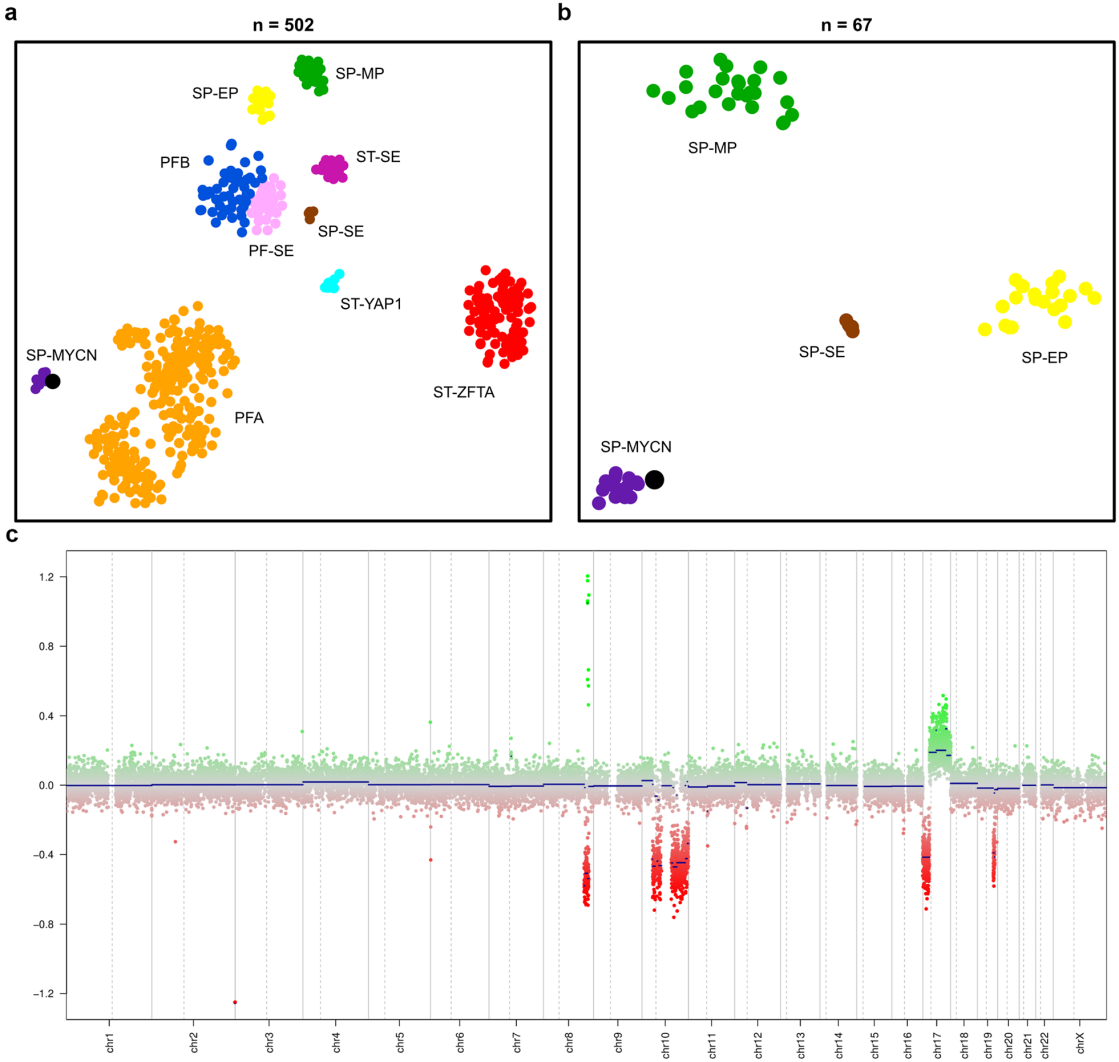
**a** t-SNE showing DNA methylation array-based clustering of the described patient case (enlarged black dot) with a reference cohort of n = 501 tumors spanning across ten established molecular EPN groups [20, 38] and **b** a subset restricted to the four established spinal EPN groups. In both analyses, the *MYC-*amplified case (enlarged black dot) clustered with the SP-MYCN group. SP-MP (spinal myxopapillary ependymoma), SP-EP (spinal anaplastic ependymoma), SP-SE (spinal subependymoma), SP-MYCN (spinal ependymoma, *MYCN*-amplified), ST-SE (supratentorial subependymoma), ST-YAP1 (supratentorial ependymoma YAP1-fused), ST-ZFTA (supratentorial ependymoma ZFTA-fused), PF-SE (posterior fossa subependymoma), PFA (posterior fossa group A), PFB (posterior fossa group B). **c** Copy number plot showing a prominent amplification at the *MYC* locus (chr8), but no alteration at the *MYCN* locus (chr2)

DNA methylation-based molecular classification was performed to assign the described patient case to one of the ten established EPN groups (SP-MP, SP-EP, SP-SE, SP-MYCN, ST-SE (supratentorial subependymoma), ST-YAP1 (supratentorial ependymoma YAP1-fused), ST-ZFTA (supratentorial ependymoma ZFTA-fused), PF-SE (posterior fossa subependymoma), PFA (posterior fossa group A), PFB (posterior fossa group B)). Unsupervised clustering with a reference cohort of 501 methylation profiles spanning all 10 established molecular EPN groups clearly assigned the tumor from our described patient case to the SP-MYCN group (Fig. 4a) [20, 38]. This result was confirmed in a repeated clustering restricted to reference cases of the four molecular spinal EPN groups (n = 66, Fig. 4b). Analyses of CNA plots of 52 spinal tumors predicted as SP-MYCN revealed a focal amplification of *MYCN* in 50/52 tumors, but no additional case with *MYC* amplification (Fig. 4c). Furthermore, when clustered with a cohort of ~ 80,000 DNA methylation profiles covering the entire spectrum of existing molecular CNS tumor classes, the described patient case classified with the SP-MYCN EPN subgroup. SP-MYCN will be included as a new reference group in the upcoming version (v12.3) of the Heidelberg Brain Tumor Methylation Classifier. This most recent version (v12.3) of the classifier assigned the described patient case to the SP-MYCN group with a calibrated score of 0.99, which is above the cut-off for confident class prediction (0.9) [10], and thus confirms the assignment of the case to the molecular group of SP-MYCN.

## Discussion and conclusions

This report describes a novel case of an aggressive recurrent progressive spinal cord ependymoma with histologic features of an anaplastic ependymoma harboring focal *MYC* amplification. Interestingly, DNA methylation-based classification assigned this case to the molecular group of SP-MYCN. *MYC* (8q24) encodes the c-MYC protein, a transcription factor that interacts with other proteins to regulate gene expression, including those that promote cell growth and proliferation [25, 48]. Deregulation of MYC has been shown to stimulate and maintain tumorigenesis in ex vivo models [28, 48]. *MYC* alterations are recurrently described amongst many different types of neoplasms, including pediatric brain tumors [6, 25], with amplification being most frequently reported [25, 28]. Glial and non-glial brain tumors harboring *MYC* amplification demonstrated a significantly worse prognosis [8, 22, 27, 30, 37, 42, 54].

Despite the high frequency of *MYC* gene alteration in human cancers, it has been rarely reported in ependymoma. A single individual was reported with a recurrent anaplastic ependymoma harboring an abnormal karyotype 46,XX,der(8)t(8;11)(q24;p11), − 11,add(?)t(?;11)(?;q13)) and *MYC* overexpression [13]. Notably, this tumor was not located in the spine but rather in the supratentorial region of the brain. Despite *MYC* overexpression, no evidence of *MYC* gene rearrangements nor amplification were identified [13]. Given the paucity of literature describing *MYC* alterations in ependymoma, we performed a literature review describing the clinical, histologic, and molecular features of the 26 reported spinal ependymomas with *MYCN* (2p24) amplification (Table 1 and Additional File 1: Table S1) [20, 41, 43, 49]. Similar to our described patient case, the SP-MYCN tumors had distinct growth patterns, and typically arose intradurally and extramedullary with invasion of the spinal cord. Most of the SP-MYCN tumors were located in the cervical or thoracic spine and were commonly associated with nodular metastatic spread and diffuse leptomeningeal involvement [20, 41, 43, 49]. Compared to other spinal ependymomas, SP-MYCN tumors were associated with aggressive behavior and unfavorable outcomes, despite intensive multi-modal therapies [20, 41]. In the described patient case, the histopathologic features including perivascular pseudo-rosettes, microvascular proliferation, densely cellular nests of mitotically active cells, prominent perivascular GFAP staining, sparse Olig-2 nuclear staining, a dot-like staining pattern with EMA, as well as increased Ki-67 proliferation indices were also described in prior reported examples of the molecular group SP-MYCN [20, 49]. Furthermore, loss of chromosome 10 may be a recurrent finding among this tumor subgroup, seen in 8/19 (42%) individuals with available copy number data (Additional File 1: Table S1). Our described patient case with *MYC* amplification also demonstrated segmental losses across chromosome 10q, including the tumor suppressor *PTEN*. Amplification of *MYC* in combination with disruption of *PTEN* has been shown in prostate cancer to contribute to aggressive disease and poor outcomes [23, 29]. Larger studies to assess the association of *MYC* or *MYCN* amplification with chromosome 10 loss and patient outcomes may be warranted.

In vitro and in vivo MYC inhibition has demonstrated tumor regression, across numerous tumor types [1, 11, 47]; nevertheless, direct inhibition of MYC is challenging. Thus, efforts have shifted towards targeting MYC transcriptional targets and regulatory domains [11, 25, 51, 53], including the study of BET inhibitors [2, 21], CDK inhibitors [5], mTOR inhibitors [26], Aurora A-kinase inhibitors [9, 17] and CHK1 inhibitors [40]. Additionally, histone deacetylase (HDAC) inhibitors have been shown to impede *MYC*-amplified Group 3 medulloblastoma tumor growth in vitro [14, 15, 39, 44]. Due to the focal *MYC* amplification, our patient received single-agent vorinostat, an oral HDAC inhibitor that is well tolerated in children with relapsed CNS tumors [19, 24, 31, 50], following his first relapse. However, he presented with progressive disease after seven months.

In summary, we report a unique case of an adolescent male with an aggressive spinal ependymal tumor harboring focal *MYC* amplification. DNA array-based methylation profiling confidently classified this tumor as SP-MYCN, a recently described subgroup of spinal ependymoma. Our described patient case demonstrates clinical, histologic, and molecular overlap with the newly described SP-MYCN subgroup. Thereby, we provide evidence to support the inclusion of *MYC* amplified spinal ependymoma within the molecular subgroup of SP-MYCN. Testing for *MYC* or *MYCN* gene amplification may be warranted in newly diagnosed spinal tumors to aid in tumor characterization. Future strategies should focus on investigating the efficacy of indirect MYC-targeting strategies, introducing new possibilities for improving the prognosis in patients with SP-MYCN.

## Supplementary Information

**Additional file 1**: Supplementary methods and data.

## Data Availability

The datasets generated and/or analyzed during the current study are available in the dbGaP repository (https://www.ncbi.nlm.nih.gov/gap/) accession phs001820.v1.p1., under submitter: Institute for Genomic Medicine (IGM) Clinical Laboratory, Nationwide Children's Hospital. Details are provided in the Additional File 1: Materials and Methods.
