# Supplementary material for: Clinically aggressive pediatric spinal ependymoma with novel MYC amplification demonstrates molecular and histopathologic similarity to newly described MYCN-amplified spinal ependymomas
Source: Acta Neuropathol Commun. 2021 Dec 11;9:192. doi: 10.1186/s40478-021-01296-2 (PMC8665631; doi:10.1186/s40478-021-01296-2)
Supplement: Supplementary file 1 — Additional file 1: Supplementary methods and data. [file 40478_2021_1296_MOESM1_ESM.docx]

**Clinically aggressive pediatric spinal ependymoma with novel *MYC* amplification demonstrates molecular and histopathologic similarity to newly described *MYCN-*amplified spinal ependymomas**

**Supplementary Materials and Methods**

*Patient Enrollment*

The adolescent male was enrolled as part of an Institutional Review Board (IRB) approved study (IRB17-00206) at the Institute for Genomic Medicine at Nationwide Children’s Hospital (NCH). Informed consent for comprehensive molecular analysis was provided by the patient’s parents. Peripheral blood (PB) was collected by routine venipuncture for genomic DNA extraction. Snap frozen tumor tissue from the primary and recurrent tumor resections were obtained for tumor DNA and RNA extraction.

*Histopathology Methods*

Immunohistochemistry was performed on formalin-fixed paraffin-embedded 4µm sections to evaluate morphology by hematoxylin and eosin (H&E) and expression of epithelial membrane antigen (EMA) (Cell Marque, cat#247M-98, Monoclonal E29, prediluted), Ki-67 (Cell Marque, cat#275R-18, Monoclonal SP6, prediluted), GFAP (Thermo (Neomarkers); cat#RB-087-R7, Polyclonal, prediluted), and Olig2 (Cell Marque, cat#387R-18, Monoclonal (EP112); prediluted). Stains were run on a Leica Bond (III and/or MAX) Automated Immunostainer.

*Enhanced exome sequencing*

Enhanced exome sequencing was performed on DNA extracted from peripheral blood (comparator sample) and disease-involved tissue (snap frozen tumor). Histologic assessment of a hematoxylin and eosin-stained section of tissue estimated tumor cellularity at 80% and 90% in the primary and recurrent tumor tissues, respectively. Libraries were prepared using 100ng of input DNA beginning with enzymatic fragmentation, followed by end repair, 5' phosphorylation, A-tailing, and sequencing adapter ligation using NEBNext Ultra II FS reagents (New England Biolabs, Ipswich, MA). Target enrichment by hybrid capture was performed with IDT xGen Exome Research Panel v1.0 enhanced with the xGenCNV Backbone and Cancer-Enriched Panels-Tech Access (Integrated DNA Technologies, Coralville, IA). Paired-end 151-bp reads were generated on the Illumina HiSeq 4000. Secondary analysis was performed using Churchill, a comprehensive workflow for analysis of raw reads from genome alignment through to germline and somatic variant identification [7]. Reads were aligned to the human genome reference sequence (build GRCh37) using BWA (v0.7.15). Sequence alignments were refined according to community-accepted guidelines for best practices (<https://gatk.broadinstitute.org/hc/en-us>). Duplicate sequence reads were removed using samblaster-v.0.1.22, and local realignment was performed on the aligned sequence data using the Genome Analysis Toolkit (v3.7–0). Churchill’s own deterministic implementation of base quality score recalibration was used. Germline variants were called using GATK’s HaplotypeCaller. Average sequencing coverage depth was 223X for the comparator peripheral blood sample, 269x for the primary tumor sample, and 441x for the recurrent tumor sample. Germline variants in cancer-associated genes were identified [18]. Somatic single nucleotide variation (SNV) and indel detection was performed using MuTect-2 [2]. Copy number alterations (CNA) was assessed using VarScan2 [8].

*RNA-sequencing*

In parallel with exome preparation, 500ng of snap frozen tumor RNA was subjected to DNase treatment and ribodepletion prior to using NEBNext Ultra II Directional RNA Library preparation reagent with incorporation of a 5 min fragmentation time. Paired-end 151-bp reads were generated on the Illumina HiSeq 4000, and reads were aligned to the human genome reference sequence (GRCh38) with the resultant output representing 218,915,085 and 101,169,207 uniquely mapped reads for the primary and recurrent tumor samples, respectively. RNA sequence data were processed using an ensemble approach of seven fusion callers (STARfusion (v.1.6.0) [6], MapSplice (v.2.2.1) [17], fusioncatcher (v.0.99.7c) [9], FusionMap (v.mono-2.10.9) [4], JAFFA (v.1.09) [3], CICERO (v0.3.0) [15], arriba (v1.2.0)) [16]. Fusions were assessed for biological significance if identified by at least three tools and the fusion was rare (<10% frequency) within our internal cancer cohort. Readthrough events, defined as fusions located within 200kb on same chromosome strand, outside of known cancer-associated aberrations were not evaluated further. A comprehensive literature review of the involved genes was performed to determine the putative mechanism (e.g. activation due to a fusion involving an oncogene or loss of function due to disruption of a tumor suppressor) and to identify previous reports of similar events. Transcripts per million (TPM) values were generated from paired-end RNA sequence data using Salmon with bootstrapping set to 100 [11]. Gene expression data were compared to publicly available RNA sequence data from the University of California Santa Cruz (UCSC) Treehouse Childhood Cancer Initiative (https://treehousegenomics.soe.ucsc.edu/).

*Fluorescence in situ hybridization*

Fluorescence *in situ* hybridization (FISH) analysis was performed on formalin-fixed paraffin-embedded (FFPE) tissue sections cut at 2µm to evaluate for amplification of the *MYC* and *MYCN* oncogenes. The estimated tumor content of both the primary and recurrent tumors was 90%. Each probe was co-hybridized with a differently labeled control probe, such that differently-labeled probes were directed to the *MYC* locus and to the centromere of chromosome 8 (LSI MYC, 8q24, Spectrum Orange; CEP 8, D8Z2, 8p11.1-q11.1, Spectrum Green; Abbott Molecular) were hybridized to the tissue sections. Additionally, differently-labeled probes directed to the *MYCN* locus and to the centromere of chromosome 2 (MYCN, 2p24.3, Spectrum Green; CEP2, D2Z1, 2p11.1-q11.1, Spectrum Orange; Abbott Molecular) were separately co-hybridized to the tissue section

*DNA-methylation profiling and Copy Number Plots*

Genome-wide DNA methylation profiling was performed using the Illumina Infinium HumanMethylation EPIC Kit. DNA methylation-based molecular classification was performed as previously described [1]. The CNV profile was derived from the EPIC methylation array data using the conumee Bioconductor package version 1.12.0 (<http://bioconductor.org/packages/conumee/>). Two previously reported cohorts including all ten established molecular groups were used as reference sets for unsupervised clustering [5, 10] as previously described [5].

**Table S1: Review of histopathological and molecular features of *MYC/MYCN-*amplified spinal ependymoma**

| **Reference** | **Case No.** | **WHO Grade** | **GFAP** | **Olig2** | **EMA** | **Mitotic activity** | **Perivascular rosettes** | **Necrosis** | **Copy Number Alterations** |
| --- | --- | --- | --- | --- | --- | --- | --- | --- | --- |
| This study | 1 (primary) | II | + | - | dot-like | 3-4/10hpf | + | + | *MYC* amplification  Gains: 17q11.2q25.3  Losses: 8q24.12q24.22, 10p11.23q11.21, 10q11.21q11.22, 10q11.22-q21.1, 10q23.1-23.31, 10q23.31-q24.2, 10q24.2q25.2, 10q25.2-q25.3, 10q25.3, 10q25.3q26.12, 10q26.12q26.13, 10q26.13, 10q26.13q26.3, 10q26.3, 17p13.3p11.2, 19q13.32q13.33, 19q13.33, |
| This study | 1 (first recurrence) | III | + | - | dot-like | up to 8/10 hpf | + | + | *MYC* amplification  Gains: 17q11.2q25.3  Losses: 8q24.12q24.22, 10p11.23q11.21, 10q11.21q11.22, 10q11.22-q21.1, 10q23.1-23.31, 10q23.31-q24.2, 10q24.2q25.2, 10q25.2-q25.3, 10q25.3, 10q25.3q26.12, 10q26.12q26.13, 10q26.13, 10q26.13q26.3, 10q26.3, 17p13.3p11.2, 19q13.32q13.33, 19q13.33, |
| [14] | 1 | III | + | - | dot-like | high | + | + | *MYCN* amplification  Gains: 1q21.1q44, 17p11.2q25.3  Losses: 2p25.3, 2p25.1p24.3, 2p24.2p23.3, 17p13.3p11.2 |
| [14] | 2 (first recurrence) | III | + | - | dot-like | high | + | + | *MYCN* amplification  16q22.1 (*NQ01*) amplification  Gains: 2, 4, 5, 8, 19  Losses: 16q22.1q22.2  cnLOH: 6 |
| [14] | 3 | III | + | - | dot-like | high | + | + | *MYCN* amplification  Gain: 18  Loss: 10 |
| [14] | 4 | III | + | - | dot-like | high | + | + | *MYCN* amplification  Gain: 19 |
| [5] | 1 (primary) | II | + | NA | dot-like | Ki67 3% | + | - | *MYCN* amplification |
| [5] | 1 (first recurrence) | III | + | NA | dot-like | Ki67 20% | + | - | *MYCN* amplification  Loss: 10 |
| [5] | 1 (second recurrence) | III | + | NA | dot-like | Ki67 30% | + | + | *MYCN* amplification  Loss: focal 2p |
| [5] | 1 (third recurrence) | III | + | NA | dot-like | Ki67 20% | + | + | *MYCN* amplification |
| [5] | 1 (fourth recurrence) | III | + | NA | dot-like | Ki67 30% | + | + | NA |
| [5] | 2 (primary) | III | + | NA | dot-like | Ki67 15% | + | - | *MYCN* amplification  Loss: 10 |
| [5] | 2 (first recurrence) | III | + | NA | dot-like | Ki67 15% | + | - | NA |
| [5] | 2 (second recurrence) | III | + | NA | dot-like | Ki67 25% | + | + | NA |
| [5] | 2 (third recurrence) | III | + | NA | dot-like | Ki67 25% | + | + | NA |
| [5] | 5 (primary) | III | + | NA | dot-like | Ki67 30% | + | - | *MYCN* amplification |
| [5] | 10 (primary) | III | + | NA | dot-like | Ki67 60% | + | + | *MYCN* amplification |
| [5] | 12 (primary) | III | + | NA | dot-like | Ki67 40% | + | - | *MYCN* amplification |
| [12] | 1 | III | + | NA | + | 15/10 hpf | + | + | *MYCN* amplification  Gain: 9p |
| [12] | 2 | III | + | NA | + | 16/10 hpf | + | + | *MYCN* amplification |
| [12] | 3 | III | + | NA | + | 17/10 hpf | + | - | *MYCN* amplification  Gains: 2, 4, 5, 8, 18  Losses: focal 2p |
| [12] | 4 | III | + | NA | + | 4/10 hpf | + | + | *MYCN* amplification  Gain: 9  Losses: 16, 19 |
| [12] | 5 | III | + | NA | + | 9/10 hpf | + | - | *MYCN* amplification  Gain: 14 |
| [12] | 6 | III | + | NA | + | 8/10 hpf | + | + | *MYCN* amplification  Loss: 10 |
| [12] | 7 | III | + | NA | + | 4/10 hpf | + | - | *MYCN* amplification  Gain: 14  Loss: 16 |
| [12] | 8 | III | + | NA | + | 10/10 hpf | + | + | *MYCN* amplification  Loss: 10q |
| [13] | 13 (primary) | II | NA | NA | NA | Ki67 7% | NA | NA | *MYCN* amplification  Gains: 12q24, 20, 22q  Loss; 13q14q31 |
| [13] | 13 (recurrence) | III | NA | NA | NA | Ki67 10% | NA | NA | *MYCN* amplification  Loss: 10 |
| [13] | 13 (metastasis) | III | NA | NA | NA | Ki67 15% | NA | NA | *MYCN* amplification  Loss: 10 |

*NA* not available, *GFAP* glial fibrillary acid protein, *EMA* epithelial membrane antigen, *hpf* high power fields, *cnLOH* copy neutral LOH

**
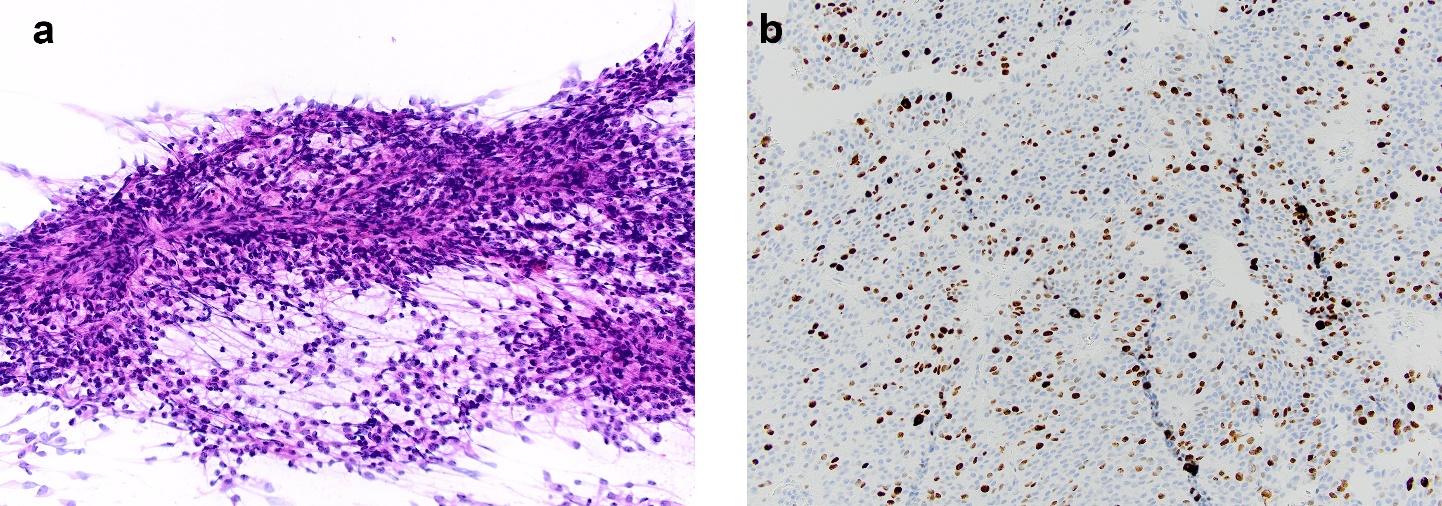
**

**Fig. S1** Histologic features of the primary tumor assessed by routine hematoxylin and eosin stain of smear/ squash cytology preparation demonstrating **(a)** perivascular rosettes as branching vascular structures with adherent angio-centric tumor cells (20x magnification) and **(b)** Ki-67 “hot spot” proliferation index reaching up to about 20% (20x magnification).

**
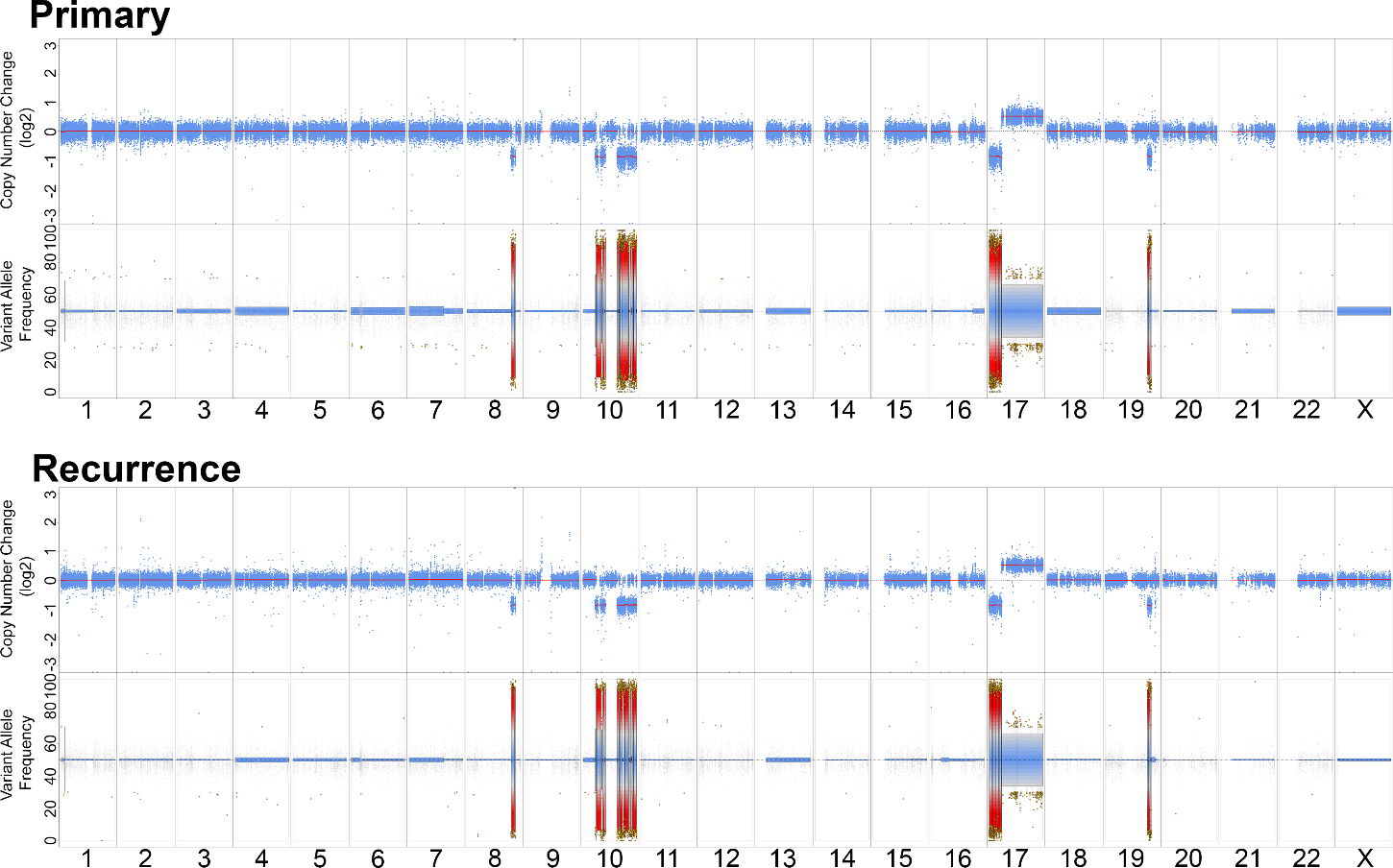
**

**Fig. S2** Genome-wide somatic copy number alterations are shown for both the primary and recurrent tumors. Top plot: Tumor copy number relative to matched normal in log2 scale. The blue points represent log2 values based on sequence depth in 100 bp windows. The red lines indicate segmented copy number calls. Bottom plot: Tumor variant allele frequency for variants that are heterozygous in the normal. The points in gold indicate a significant loss of heterozygosity. The x-axis denotes the chromosome number.

**
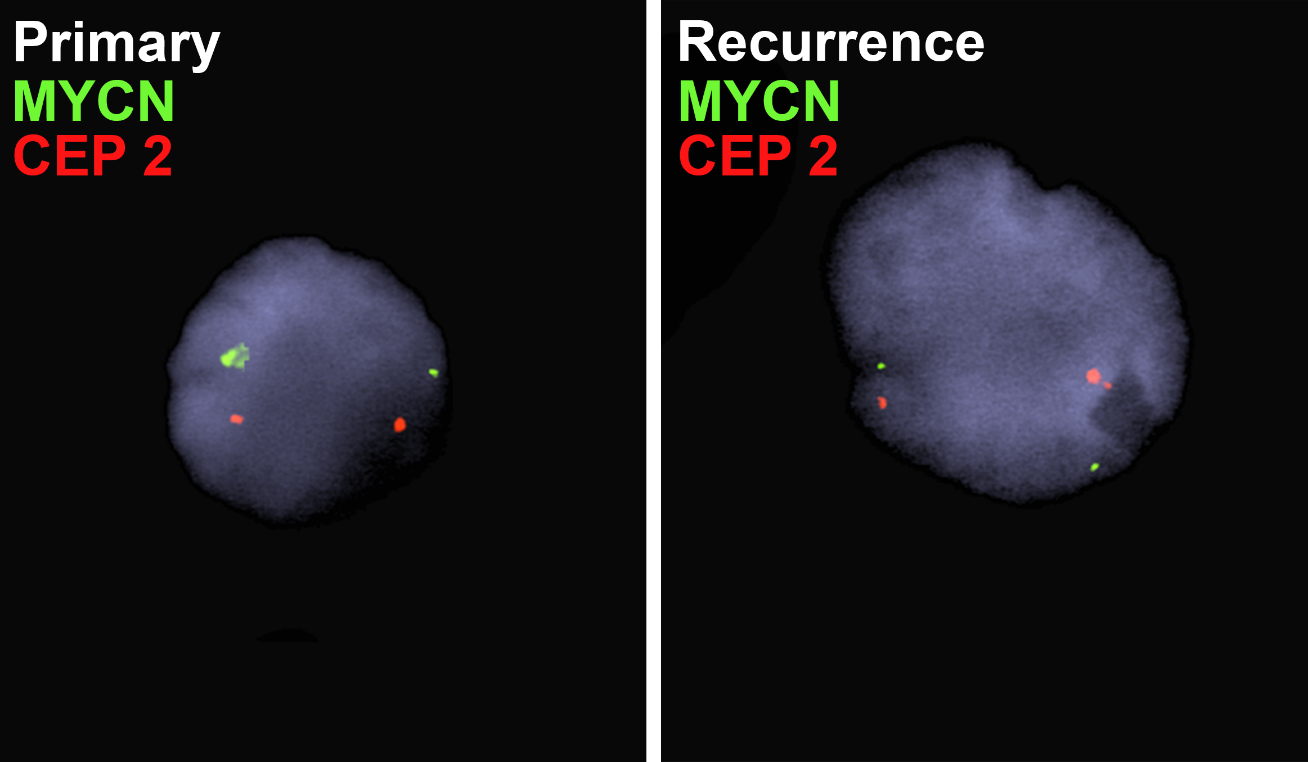
**

**Fig. S3** Fluorescence *in situ* hybridization (FISH) of the *MYCN* locus. *MYCN* (green) demonstrates a normal diploid complement with two signals for the *MYCN* locus and two signals of chromosome 2 centromere (red).

**References**

1 Capper D, Jones DTW, Sill M, Hovestadt V, Schrimpf D, Sturm D, Koelsche C, Sahm F, Chavez L, Reuss DE et al (2018) DNA methylation-based classification of central nervous system tumours. Nature 555: 469-474 <https://doi.org/10.1038/nature26000>

2 Cibulskis K, Lawrence MS, Carter SL, Sivachenko A, Jaffe D, Sougnez C, Gabriel S, Meyerson M, Lander ES, Getz G (2013) Sensitive detection of somatic point mutations in impure and heterogeneous cancer samples. Nat Biotechnol 31: 213-219 <https://doi.org/10.1038/nbt.2514>

3 Davidson NM, Majewski IJ, Oshlack A (2015) JAFFA: High sensitivity transcriptome-focused fusion gene detection. Genome Med 7: 43 <https://doi.org/10.1186/s13073-015-0167-x>

4 Ge H, Liu K, Juan T, Fang F, Newman M, Hoeck W (2011) FusionMap: detecting fusion genes from next-generation sequencing data at base-pair resolution. Bioinformatics 27: 1922-1928 <https://doi.org/10.1093/bioinformatics/btr310>

5 Ghasemi DR, Sill M, Okonechnikov K, Korshunov A, Yip S, Schutz PW, Scheie D, Kruse A, Harter PN, Kastelan M et al (2019) MYCN amplification drives an aggressive form of spinal ependymoma. Acta Neuropathol 138: 1075-1089 <https://doi.org/10.1007/s00401-019-02056-2>

6 Haas BJ, Dobin A, Li B, Stransky N, Pochet N, Regev A (2019) Accuracy assessment of fusion transcript detection via read-mapping and de novo fusion transcript assembly-based methods. Genome Biol 20: 213 <https://doi.org/10.1186/s13059-019-1842-9>

7 Kelly BJ, Fitch JR, Hu Y, Corsmeier DJ, Zhong H, Wetzel AN, Nordquist RD, Newsom DL, White P (2015) Churchill: an ultra-fast, deterministic, highly scalable and balanced parallelization strategy for the discovery of human genetic variation in clinical and population-scale genomics. Genome Biol 16: 6 <https://doi.org/10.1186/s13059-014-0577-x>

8 Koboldt DC, Zhang Q, Larson DE, Shen D, McLellan MD, Lin L, Miller CA, Mardis ER, Ding L, Wilson RK (2012) VarScan 2: somatic mutation and copy number alteration discovery in cancer by exome sequencing. Genome Res 22: 568-576 <https://doi.org/10.1101/gr.129684.111>

9 Nicorici D, Satalan M, Edgren H, Kangaspeska S, Murumagi A, Kallioniemi O, Virtanen S, Kilkku O (2014) FusionCatcher - a tool for finding somatic fusion genes in paired-end RNA-sequencing data. bioRxiv: 011650 <https://doi.org/10.1101/011650>

10 Pajtler Kristian W, Witt H, Sill M, Jones David TW, Hovestadt V, Kratochwil F, Wani K, Tatevossian R, Punchihewa C, Johann P et al (2015) Molecular Classification of Ependymal Tumors across All CNS Compartments, Histopathological Grades, and Age Groups. Cancer Cell 27: 728-743 <https://doi.org/10.1016/j.ccell.2015.04.002>

11 Patro R, Duggal G, Love MI, Irizarry RA, Kingsford C (2017) Salmon provides fast and bias-aware quantification of transcript expression. Nat Methods 14: 417-419 <https://doi.org/10.1038/nmeth.4197>

12 Raffeld M, Abdullaev Z, Pack SD, Xi L, Nagaraj S, Briceno N, Vera E, Pittaluga S, Lopes Abath Neto O, Quezado M et al (2020) High level MYCN amplification and distinct methylation signature define an aggressive subtype of spinal cord ependymoma. Acta Neuropathol Commun 8: 101 https://doi.org/10.1186/s40478-020-00973-y

13 Scheil S, Bruderlein S, Eicker M, Herms J, Herold-Mende C, Steiner HH, Barth TF, Moller P (2001) Low frequency of chromosomal imbalances in anaplastic ependymomas as detected by comparative genomic hybridization. Brain Pathol 11: 133-143 <https://doi.org/10.1111/j.1750-3639.2001.tb00386.x>

14 Swanson AA, Raghunathan A, Jenkins RB, Messing-Junger M, Pietsch T, Clarke MJ, Kaufmann TJ, Giannini C (2019) Spinal Cord Ependymomas With MYCN Amplification Show Aggressive Clinical Behavior. J Neuropathol Exp Neurol 78: 791-797 https://doi.org/10.1093/jnen/nlz064

15 Tian L, Li Y, Edmonson MN, Zhou X, Newman S, McLeod C, Thrasher A, Liu Y, Tang B, Rusch MC et al (2020) CICERO: a versatile method for detecting complex and diverse driver fusions using cancer RNA sequencing data. Genome Biol 21: 126 https://doi.org/10.1186/s13059-020-02043-x

16 Uhrig S, Ellermann J, Walther T, Burkhardt P, Frohlich M, Hutter B, Toprak UH, Neumann O, Stenzinger A, Scholl C et al (2021) Accurate and efficient detection of gene fusions from RNA sequencing data. Genome Res 31: 448-460 https://doi.org/10.1101/gr.257246.119

17 Wang K, Singh D, Zeng Z, Coleman SJ, Huang Y, Savich GL, He X, Mieczkowski P, Grimm SA, Perou CM et al (2010) MapSplice: accurate mapping of RNA-seq reads for splice junction discovery. Nucleic Acids Res 38: e178 https://doi.org/10.1093/nar/gkq622

18 Zhang J, Walsh MF, Wu G, Edmonson MN, Gruber TA, Easton J, Hedges D, Ma X, Zhou X, Yergeau DA et al (2015) Germline Mutations in Predisposition Genes in Pediatric Cancer. N Engl J Med 373: 2336-2346 https://doi.org/10.1056/NEJMoa1508054
